# Erjingwan and Alzheimer’s disease: research based on network pharmacology and experimental confirmation

**DOI:** 10.3389/fphar.2024.1328334

**Published:** 2024-04-29

**Authors:** Yuya Xu, Jian Zhang, Xuling Li

**Affiliations:** ^1^ Department of Neurology, The Fourth Affiliated Hospital, Harbin Medical University, Harbin, Heilongjiang, China; ^2^ School of Basic Medicine, Heilongjiang University of Chinese Medicine, Harbin, Heilongjiang, China

**Keywords:** Erjingwan, Alzheimer’s disease, network pharmacology, hippocampus, AGEs/RAGE/NF-κB pathway

## Abstract

**Background:**

Alzheimer’s disease (AD), a challenging neurodegenerative condition, has emerged as a significant global public health concern. The Chinese medicine decoction Erjingwan (EJW) has shown promising efficacy in AD treatment, though its mechanism remains unclear.

**Objective:**

This study aims to elucidate the mechanism by which EJW treats AD through network pharmacology analysis and *in vivo* experiments.

**Methods:**

We identified EJW’s components using the Traditional Chinese Medicine Systems Pharmacology (TCMSP) database and determined AD-related targets from various databases. A network comprising herbs-compounds-targets was established, and EJW’s core targets were ascertained through protein-protein interaction (PPI) analysis. This study assessed the cognitive abilities of APP/PS1 mice using Morris water mazes and Y mazes, in addition to analyzing blood samples for triglyceride (TG), total cholesterol (TC), low-density lipoprotein (LDL), and high-density lipoprotein (HDL) levels. Brain tissues were examined histologically with HE staining, Nissl staining, and immunohistochemistry (IHC) for amyloid β-protein (Aβ) detection. Superoxide dismutase (SOD), reactive oxygen species (ROS), Interleukin-1β (IL-1β), and Interleukin-6 (IL-6) levels in the hippocampal region were measured by ELISA. mRNA expression of apolipoprotein A-I (APOA-I), apolipoprotein B (APOB), apolipoprotein E4 (APOE4), advanced glycation end products (AGE), the receptor for AGE (RAGE), and nuclear factor kappa-B (NF-κB) was evaluated by quantitative PCR (q-PCR). Western blotting was used to detect the expression of AGE, RAGE, NF-κB, and Tau protein.

**Results:**

Screening identified 57 chemical components and 222 potential targets of EJW. Ten core targets for AD treatment were identified, with enrichment analysis suggesting EJW’s effects are related to lipid metabolism and AGEs/RAGE pathways. EJW enhanced learning and memory in APP/PS1 mice, protected neuronal structure in the hippocampal region, reduced Aβ deposition, and altered levels of TG, TC, LDL, IL-1β, and IL-6, and the expression of APOE4, AGEs, RAGE, NF-κB, and Tau protein, while increasing SOD, APOA-I, and APOB mRNA expression.

**Conclusion:**

The study identified four core components of EJW—iosgenin, baicalein, beta-sitosterol, quercetin—and ten core targets including AKT1, IL6, VEGFA, TP53, CASP3, for treating AD. Experimental results demonstrate EJW’s capacity to modulate lipid profiles, reduce pathological markers such as Aβ_1-42_, Tau, IL-6, IL-1β, reactive oxygen species, SOD, and enhance cognitive functions in APP/PS1 mice, potentially through inhibiting the AGEs/RAGE/NF-κB pathway.

## 1 Introduction

Alzheimer’s disease (AD) is a degenerative disorder of the central nervous system characterized by progressive cognitive and behavioral impairments (Scheltens et al., 2021). AD constitutes the majority of dementia cases, representing 75% of all instances (O Neill and Kennelly, 2021). Currently, over 50 million individuals worldwide suffer from AD, a figure projected to triple by 2050 (Twarowski and Herbet, 2023). In China, a rapidly aging population presents a significant challenge, with estimates suggesting 30.03 million AD patients by 2050 (Wang et al., 2019).

AD pathology is marked by the excessive aggregation of amyloid β-protein (Aβ) and hyperphosphorylation of tau protein, leading to neuronal loss and cell death. The cleavage of amyloid precursor protein (APP) by β-secretase and γ-secretase produces neurotoxic Aβ_25-35_ and Aβ_1-42_ peptides, which activate immune cells in the central nervous system, inducing neuroinflammation (Thal et al., 2002; Sun et al., 2017; Dhapola et al., 2021). This activation predominantly occurs as Aβ binds to microglia and astrocytes, triggering the release of inflammatory factors such as tumor necrosis factor-α (TNF-α), nuclear factor-κB (NF-κB), and interleukin-6 (IL-6) (Calsolaro and Edison, 2016). Additionally, Aβ promotes oxidative stress by disrupting the balance between oxidation and antioxidation, damaging mitochondria and impairing ATP synthesis (Ionescu-Tucker and Cotman, 2021). Excessive accumulation of Aβ impairs mitochondria, restricting mitochondrial fusion and enhancing mitochondrial fission, thereby obstructing the synthesis of adenosine triphosphate (ATP). A decline in antioxidant enzymes, such as superoxide dismutase (SOD), glutathione peroxidase (GPx), glutaredoxins, and thioredoxins, results in elevated levels of ROS (Tönnies and Trushina, 2017). Consequently, this inhibition prevents synapses from releasing neurotransmitters, leading to memory loss. Moreover, Aβ accumulation elevates tau protein phosphorylation (Zhang et al., 2021a). Tau,a crucial microtubule (MT)-binding protein, undergoes multi-site phosphorylation by various enzymes, including cyclin-dependent kinase-5 (CDK-5), calcium/calmodulin-dependent protein kinase II, and glycogen synthase kinase-3β (GSK-3β), which regulates tau’s binding to MTs. However, Aβ facilitates the overactivation of GSK-3β and CDK-5, causing tau hyperphosphorylation and detachment from MTs, leading to the formation of neurofibrillary tangles (NFTs) and, ultimately, to the onset of AD (Bloom, 2014). Additionally, Aβ is closely linked to AD development. Recent research has associated imbalances in intestinal flora, dysfunction of the cholinergic system, and anomalies in apoptosis and autophagy with Aβ misfolding. Despite advancements, a definitive cause of AD remains unidentified, and the efficacy of targeted therapies for the aforementioned pathological processes has been less than ideal (Breijyeh and Karaman, 2020; Kesika et al., 2021).

The treatment of AD is predicated on the belief that its primary cause is the diminution of kidney essence in the elderly, which fails to nourish the brain, resulting in memory decline. Consequently, the core approach focuses on enhancing cognitive and memory functions and augmenting kidney essence to combat AD. Erjingwan (EJW), a revered traditional Chinese medicine formula, originates from the ancient text “Sheng Ji Zong Lu”. This formula comprises two herbal components: *Polygonatum sibiricum Redouté* (Chinesse name:Huang Jing) and *Lycium chinense Mill* (Chinesse name:Gou Qi), known for their benefits in kidney nourishment, essence replenishment, and aging delay (Yang et al., 2021). Furthermore, contemporary pharmacological investigations have confirmed that Huang Jing and Gou Qi enhance learning and memory in AD models, reduce Aβ deposition in the brain, and mitigate aging (Ye et al., 2015; Yao et al., 2018; Luo et al., 2022; Zhang et al., 2022). Despite EJW’s proven efficacy in AD treatment, its mechanistic basis remains elusive.

**FIGURE 1:**
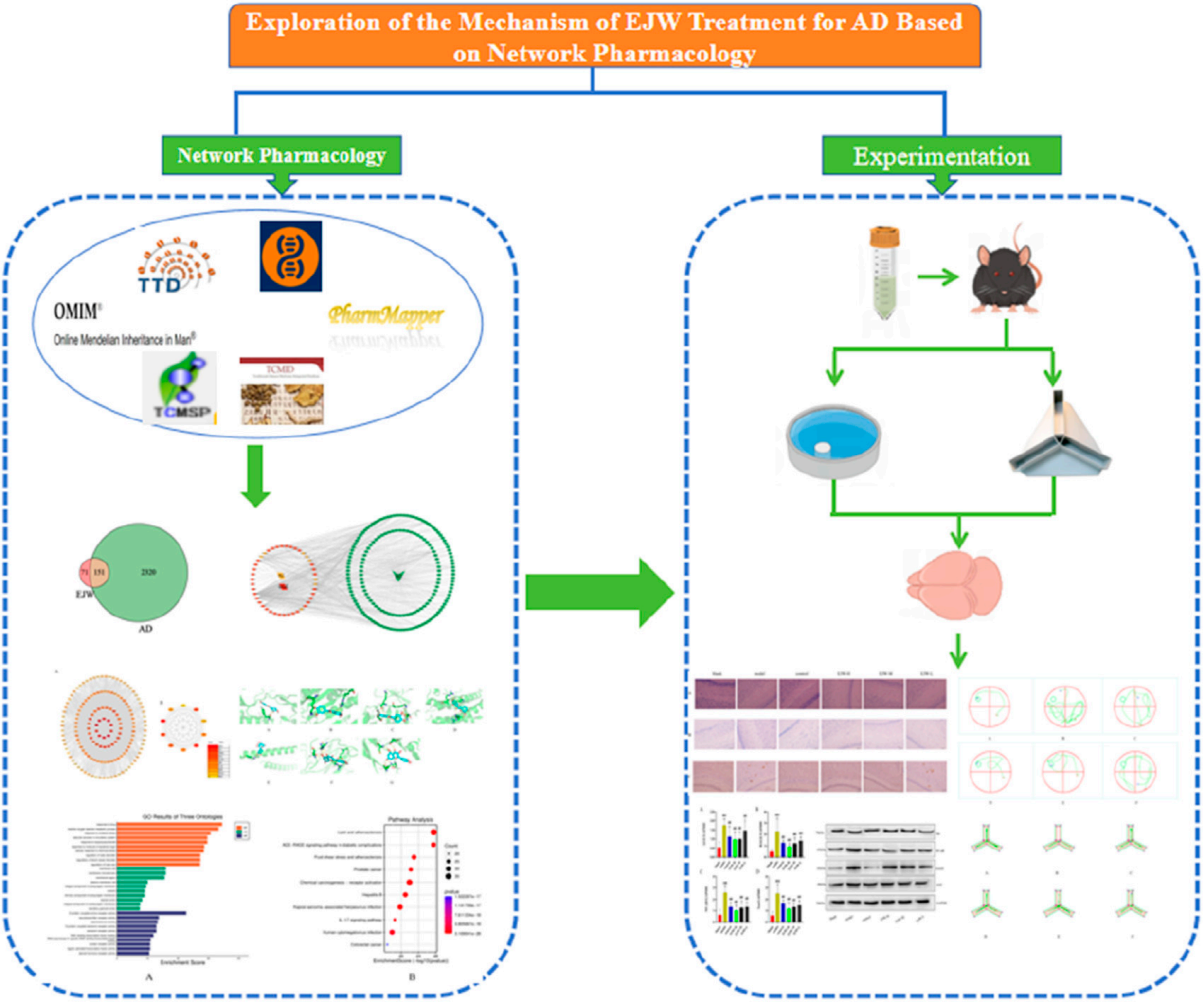
Workflow for studying the mechanisms of EJW in AD treatment.

Introduced by Hopkins, the concept of network pharmacology aims to address the complexities of drug-body interactions (Hopkins, 2007). Given traditional Chinese medicine’s broad-spectrum efficacy and complex mechanisms, pinpointing precise intervention strategies poses a challenge. Network pharmacology, utilizing the Traditional Chinese Medicine Systems Pharmacology Database (TCMSP), facilitates the identification of compounds, prediction of targets, and construction of a comprehensive “component-target-disease” network, enhancing the development and application of traditional Chinese medicines (Jiashuo et al., 2022). This study’s objective is to unravel the biological mechanisms underlying EJW’s effectiveness in AD treatment, identify its active components and targets, and corroborate its impact through *in vivo* experiments, providing theoretical justification for EJW’s therapeutic potential against AD (Figure 1).

## 2 Methods

### 2.1 Chemical selection and target prediction

The chemical components of EJW were sourced from the TCMSP (http://tcmspw.com/tcmsp.php) and the Traditional Chinese Medicine Integrated Database (TCMID) (http://www.niegabionet.org/tcmid/). Screening criteria were set for oral availability (OB) ≥30% and drug-likeness (DL) ≥0.18%. The extracted chemical composition data were entered into PharmMapper (http://www.lilab-ecust.cn/pharmmapper/), and gene names were standardized using the UniProt database (http://www.uniprot.org/) (Shang et al., 2023).

### 2.2 Disease target screening

Relevant Alzheimer’s disease targets were compiled from the Online Mendelian Inheritance in Man (OMIM) database (http://www.omim.org), the Therapeutic Target Database (TTD) (http://bidd.nus.edu.sg/BIDD-Databases/TTD/TTD.asp), GeneCards (https://www.genecards.org), and DrugBank (https://www.drugbank.com) (Niu et al., 2022).

### 2.3 Protein-protein interaction (PPI) analysis

Shared target genes between EJW components and AD were uploaded to the STRING database (https://string-db.org/) with the species set to “Homo sapien”. The minimum interaction threshold was defined as " Highest confidence>0.9". Default settings were maintained for other parameters, and the output was saved in TSV format. This file was then imported into Cytoscape 3.9.1, where isolated nodes were removed. The CytoHubba plugin identified the top 10 core targets, facilitating the creation of a visual PPI network (Lin et al., 2023).

### 2.4 Construction of “herbs-compounds-targets”network and enrichment analysis

To map the “herbs-compounds-targets” relationship, common target genes of EJW components and AD were imported into Cytoscape3.9.1. Analysis of these targets through Gene Ontology (GO) and Kyoto Encyclopedia of Genes and Genomes (KEGG) enrichment analysis was conducted, setting *p* < 0.05 as the threshold to exclude cellular components (CC), biological processes (BP), molecular functions (MF), and enrichment pathways. R language was used for visualization (Zhao et al., 2023).

### 2.5 Molecular docking

AutoDock Vina software (http://vina.scripps.edu/) was employed for docking chemical components with disease targets. To prepare the ligands and proteins, target proteins were retrieved from the PDB database (https://www.rcsb.org/), undergoing preprocessing to remove water molecules, modify amino acids, optimize energy, and adjust force field parameters. Ligand structures were sourced from the PubChem database (https://pubchem.ncbi.nlm.nih.gov/) to ensure low-energy conformations. Subsequently, molecular docking was conducted using PyRx (https://pyrx.sourceforge.io/), with visualizations provided by PyMOL (https://pymol.org/2/). Discovery Studio 2020 Client (https://discover.3ds.com/discovery-studio-visualizer-download) was utilized for analyzing 2D diagrams (Huang et al., 2023).

### 2.6 Drugs and reagents

The EJW herbs were supplied by the First Affiliated Hospital of Heilongjiang University of Traditional Chinese Medicine, with authentication by Professor Sun Huifeng. In preparation, 1400 g of both *Polygonatum sibiricum Redouté* and *Lycium chinense Mill* were ground and soaked in distilled water (1:5 W/V) for 30 min, followed by extraction through boiling twice at 100°C for 1 h each time. Concentrate the mixture to achieve a density of 1.2 g mL^-1^ and store at −4°C post-concentration. Donepezil (Weicai (China) Pharmaceutical Co., Ltd.), tissue fixation solution (Dalian Meilun Company), hematoxylin-eosin solution (Beijing Regen Company), methylene blue (Beijing Solarbio Company), xylene (Tianjin Fuyu Fine Chemical Co., Ltd.), ethanol (Tianjin Tianli Chemical Reagent Co., Ltd.), PBS (Germany Sigma-Aldrich Company), Anti-Aβ1-42 (Abcam, USA), ELISA test kit (Abcam, USA), DEPC (Sigma, USA), ChamQ Universal SYBR qPCR Master Mix (Nanjing Vazyme Company), HiScript II Q RT SuperMix for qPCR (+gDNA wiper) (Nanjing Vazyme Company), RIPA lysis buffer (Beijing Beyotime Company), BCA assay kit (Beijing Solarbio Company), SDS (Sigma, USA), Page ruler prestained protein ladder (Thermo Fisher Scientific, USA), anti-AGE, anti-RAGE, Anti-NF-κB, Anti-TNF alpha, Anti-Tau and GAPDH antibody (Abcam, USA).

### 2.7 Instruments and equipments

The laboratory utilized a range of specialized equipment, including the Microplate reader HM-SY96S (Shandong Hengmei Electronic Technology Co., Ltd.), Electrophoresis instrument of type DYCZ-24DN (Beijing Liuyi Biotechnology Co., Ltd.), Microscope E100 (Nikon Corporation, Japan), Low temperature centrifuge TGL-16M (Changsha Xiangyi Testing Equipment Co., Ltd.), Automated tissue embedding system (HistoCore Arcadia), Microtome with a fully automatic cutting system (Leica Company, Germany), PCR system Veriti ABI (Thermo Fisher Scientific, USA), Tissue grinder Tissuelyser-24 (Shanghai Jingxin Industrial Development Co., Ltd.), and Fully automatic luminescence imager Tanon-5200 (Shanghai Tanon Technology Co., Ltd.), along with another Electrophoresis system A-15907-2213 (Shanghai Tanon Technology Co.).

### 2.8 Animal grouping and drug administration

Fifty 6-month-old male APP/PS1 mice (SCXK (su) 2016-0010) were acquired from Cavens Experimental Animal (Changzhou) Co., Ltd., with an average weight of 25.62 ± 4.39 g, and were randomly assigned to one of six groups: blank, model, control, EJW-H, EJW-M, and EJW-L, with ten mice per group. Additionally, ten C57BL/6 mice, weighing 23.51 ± 3.26 g, were sourced from Liaoning Changsheng Biotechnology Co., Ltd. for use as a blank group. Each mouse group was housed in individual cages within a breeding room equipped with an independent ventilation system, and drug treatment commenced following a 7-day acclimatization period (Zhong et al., 2019). The dosages for the EJW-treated groups were based on prior research, set at 9, 4.5 and 2.25 g kg^-1^·d^-1^, respectively. The control group received donepezil hydrochloride (0.5 mg kg^-1^). For 30 days, mice in the model and blank groups were administered normal saline via gavage at 0.01 mL/g.

### 2.9 Behavioral experiment

The learning and memory capabilities of APP/PS1 mice were assessed through the Morris water maze and Y maze tests. In the Morris water maze, after 1 day of adaptive training, all the mice were placed facing outward in each quadrant to record the time taken to locate the hidden platform, termed the escape latency. This phase lasted 5 days, with the sixth day involving removal of the platform to observe free swimming and recording time spent in the target quadrant and platform crossings (Bromley-Brits et al., 2011). The Y maze test commenced with an adaptation phase, during which mice were exposed to two open arms, with the closed arm introduced as a new arm the following day. Mice were allowed to explore from a designated starting arm, and their entries into the new arm, duration of stay, and spontaneous alternation were documented (Kraeuter et al., 2019).

### 2.10 HE, Nissl staining, and immunohistochemistry (IHC)

Following anesthesia, mice underwent cardiac perfusion. Their brain tissues were extracted on ice, fixed in paraformaldehyde for 6 h, dehydrated through alcohol gradients, permeabilized with xylene, and embedded in paraffin. Tissue sections, after being cut and de-waxed, were treated with xylene and graded alcohols, then rinsed under running water. For HE staining, sections were immersed in hematoxylin and eosin, while others underwent Nissl staining with toluidine blue. Remaining sections were prepared for immunohistochemistry by incubation in phosphate-buffered saline (PBS), antigen retrieval with EDTA, and quenching in 3% hydrogen peroxide. Sections were then incubated with Anti-Aβ_1-42_ (1; 1000, cat no. ab201061) primary antibody at 4°C for 16 h. Following rewarming, sections were washed with PBS, treated with secondary antibodies for 20 min at 37°C, developed with DAB, counterstained with hematoxylin, differentiated, dehydrated, permeabilized, and sealed (Hussaini et al., 2023).

### 2.11 Biochemical detection

Blood was collected from the orbital veins of fasted mice, and serum was separated by centrifugation at 3000rpm for 15 min, then stored in frozen tubes for TG, TC, LDL, and HDL analysis.

### 2.12 ELISA

Post-anesthesia, mice were euthanized, and the hippocampus was isolated. A 30 mg sample of hippocampal tissue was stored in an EP tube with a 1 mg:5 μL ratio of pre-chilled PBS. The sample, along with steel beads, was homogenized twice for 2 min in a pre-chilled grinder, then centrifuged at 12,000 rpm for 10 min. The supernatant was subjected to Anti-SOD (cat no. ab80946), Anti-ROS (cat no. ab238535), Anti-IL-1β (cat no. ab2105) and Anti-IL-6 (cat no. ab9324) ELISA assays according to kit instructions. An ELISA reader measured absorbance to determine the expression levels of SOD, ROS, IL-1β, and IL-6 in the hippocampus.

### 2.13 RT-PCR

RT-PCR was conducted to detect the expression of APOA-I, APOB, APOE4, AGEs, RAGE, and NF-κB mRNA in mouse brain tissue (Table 1). Initially, 1 mL of Trizol reagent was added to the tissue, thoroughly mixed, and transferred to a 1.5 mL RNase-Free centrifuge tube, where it was left at room temperature for 5 min. Following this, 200 μL of chloroform was added, mixed by inversion, and centrifuged at 4°C for 15 min. Isopropanol, in a 1:1 ratio with the supernatant, was then added, mixed by inversion, and left at room temperature for 10 min. Afterward, 1 mL of 75% ethanol was added, and the tube was shaken to resuspend the precipitate before centrifugation at 4°C for 5 min. The RNA pellet was air-dried, dissolved in 20 μL of diethylpyrocarbonate (DEPC) water, and stored at −80°C. RNA concentration and purity were measured using a microspectrophotometer, followed by reverse transcription to synthesize cDNA, which was then quantified to 200 ng/μL. For PCR, n+1 mixtures were prepared in 1.5 mL centrifuge tubes, with 1 μL of cDNA template added to each, briefly centrifuged and mixed, followed by amplification using a fluorescence quantitative PCR instrument.

**TABLE 1:** Primer sequence.

Interest genes	Primer sequences (5′-3′)	SIZE
APOA-I	F:5′-GGG ACA CTC TGG GTT CAA-3′	107bp
	R:5′-CTC GTT TCT CAG CCA ATC T-3′	
APOB	F:5′-CCTGCCATGGGAAACATTAC-3′	150bp
	R:5′-TGCAGTGCATCAATGACAGA-3′	
APOE4	F:5′-CCCAGGTCACCCAGGAACT-3′	56bp
	R:5′-AGTTCCGATTTGTAGGCC-3′	
AGE	F:5′-CCCAATGGTTCACTCCTCCTT-3′	223bp
	R:5′-AGAAAGTGGCTCGAGGTTGA-3′	
RAGE	F:5′-CCTTCCT-CGGCACAGACC-3′	104bp
	R:5′-TTCCACCTTCAGGCTCAACC-3′	
NF-κB	F:5′-AATGGCTACACAGGACCAGGAAC-3′	294bp
	R:5′-TGGCTAATGGCTTGCTCCAG-3′	
GAPDH	F:5′-ACAGCAACAGGGTGGTGGAC-3′	496bp
	R:5′-TTTGAGGGTGCAGCGAACTT-3′	

### 2.14 Western blot

For Western blot analysis, 100 mg of brain tissue from each group was minced on ice. The samples were lysed using RIPA buffer, homogenized for 30 min, and then transferred to a 1.5mL centrifuge tube. After centrifugation at 12,000 rpm for 5 min at 4°C to clarify, the protein concentration was determined. The proteins were denatured by adding 4×SDS loading buffer and heating in a 100°C water bath for 5 min. A total of 50 μg of protein per sample was subjected to SDS-PAGE, followed by transfer to a PVDF membrane. The membrane was blocked with 5% BSA for 1 h before incubation with primary antibodies against Anti-AGE (1:1000, cat no. ab23722), Anti-RAGE (1:1000, cat no. ab216329), Anti-NF-κB p50 (1:1000, cat no. ab220803), Anti-Tau phospho S396 (1:1000, cat no. ab32057), and GAPDH (1:10,000, cat no. ab8245) at 4°C overnight. After washing, the membrane was incubated with a secondary antibody (1:2000) at 37°C for 1.5 h, washed six times, and developed with ECL. Images were captured in a gel imaging analysis system, and the gray value ratio of the target protein to the internal reference was used to quantify relative protein expression levels for statistical analysis.

### 2.15 Statistic analysis

Data were analyzed using SPSS 23.0 software and are expressed as mean ± standard deviation (mean ± SD). Intergroup comparisons were conducted via one-way analysis of variance (ANOVA), while repeated measures ANOVA was utilized for escape latency data. A significance threshold of *p* < 0.05 was established to identify statistically significant differences.

## 3 Results

### 3.1 Chemical composition and disease target information of EJW

**FIGURE 2:**
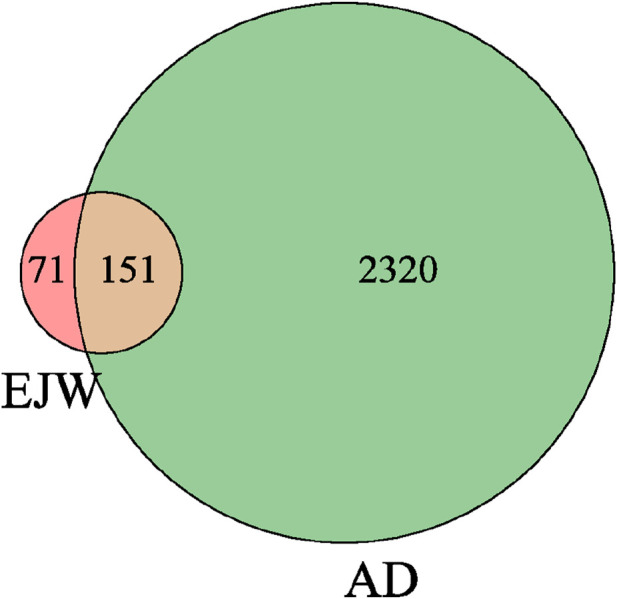
Venn diagram of drug components and disease targets.

Initial screening of EJW’s herbal components yielded 57 compounds, with 12 derived from *Polygonatum sibiricum Redouté* and 45 from *Lycium chinense Mill*. Of these, four components from *Polygonatum sibiricum Redouté* lacked corresponding target information. Further analysis identified 292 potential target proteins for these compounds, including 89 targets associated with *Polygonatum sibiricum Redouté* and 203 with *Lycium chinense Mill*, with 70 targets common to both. In total, 2471 AD-related targets were sourced from various disease databases. Comparing the herbal components' potential targets with those related to AD highlighted 151 potential targets for EJW’s efficacy against AD, as depicted in a Venn diagram (Figure 2).

### 3.2 Analysis of “herbs-compounds-targets” interaction network

**FIGURE 3:**
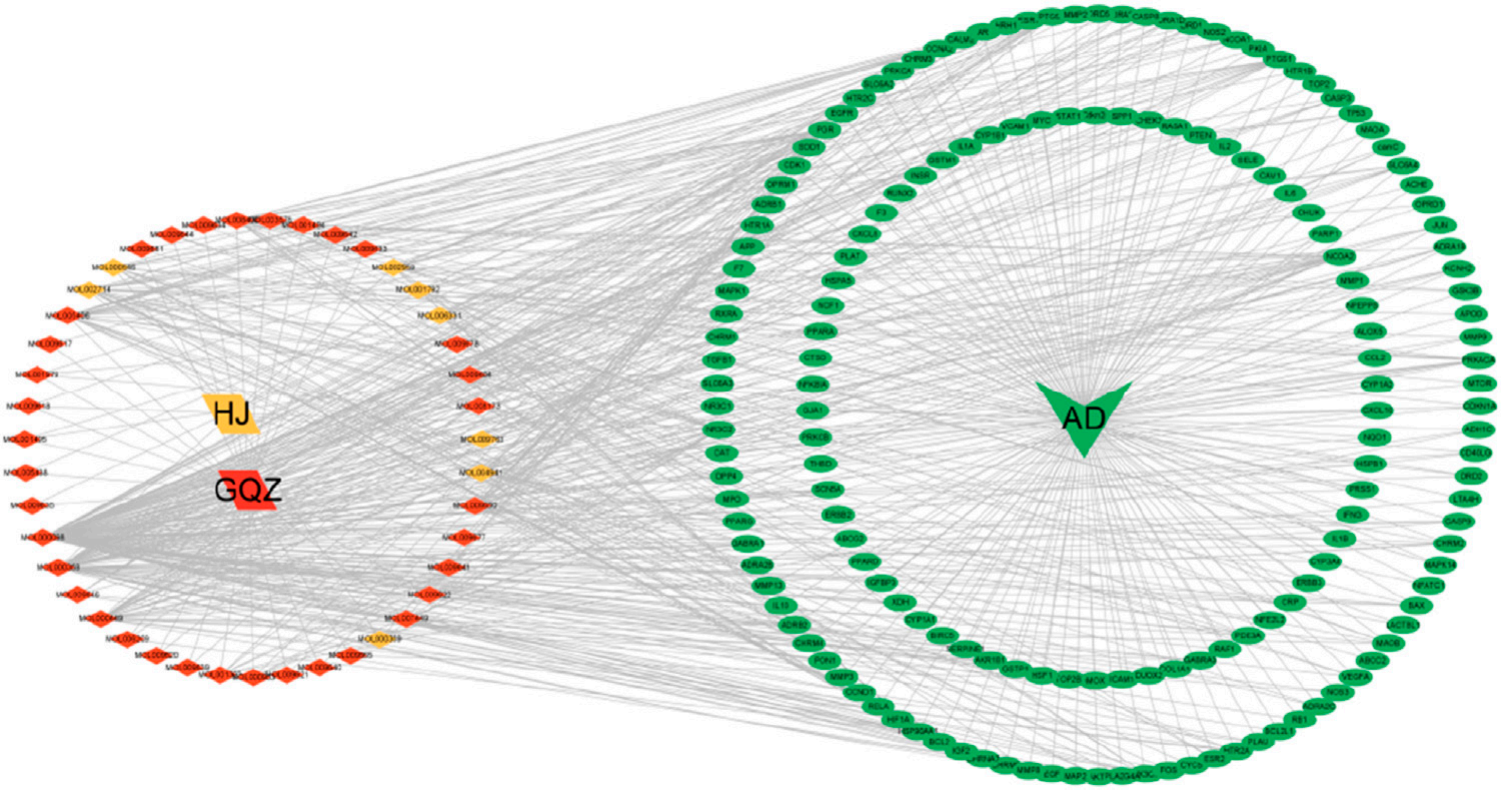
“Herbs-compounds-targets” interaction network.

By docking the identified targets of EJW’s components with AD targets, an “herbs-compounds-targets” interaction network was constructed (Figure 3). Notably, component MOL000098 from *Lycium chinense Mill* berries demonstrated the highest number of interactions with AD targets. Additionally, components MOL000358, MOL000449, MOL005406, and MOL002714 from *Polygonatum sibiricum Redout* were identified as potentially significant active ingredients for EJW’s action against AD.

### 3.3 PPI network analysis

**FIGURE 4:**
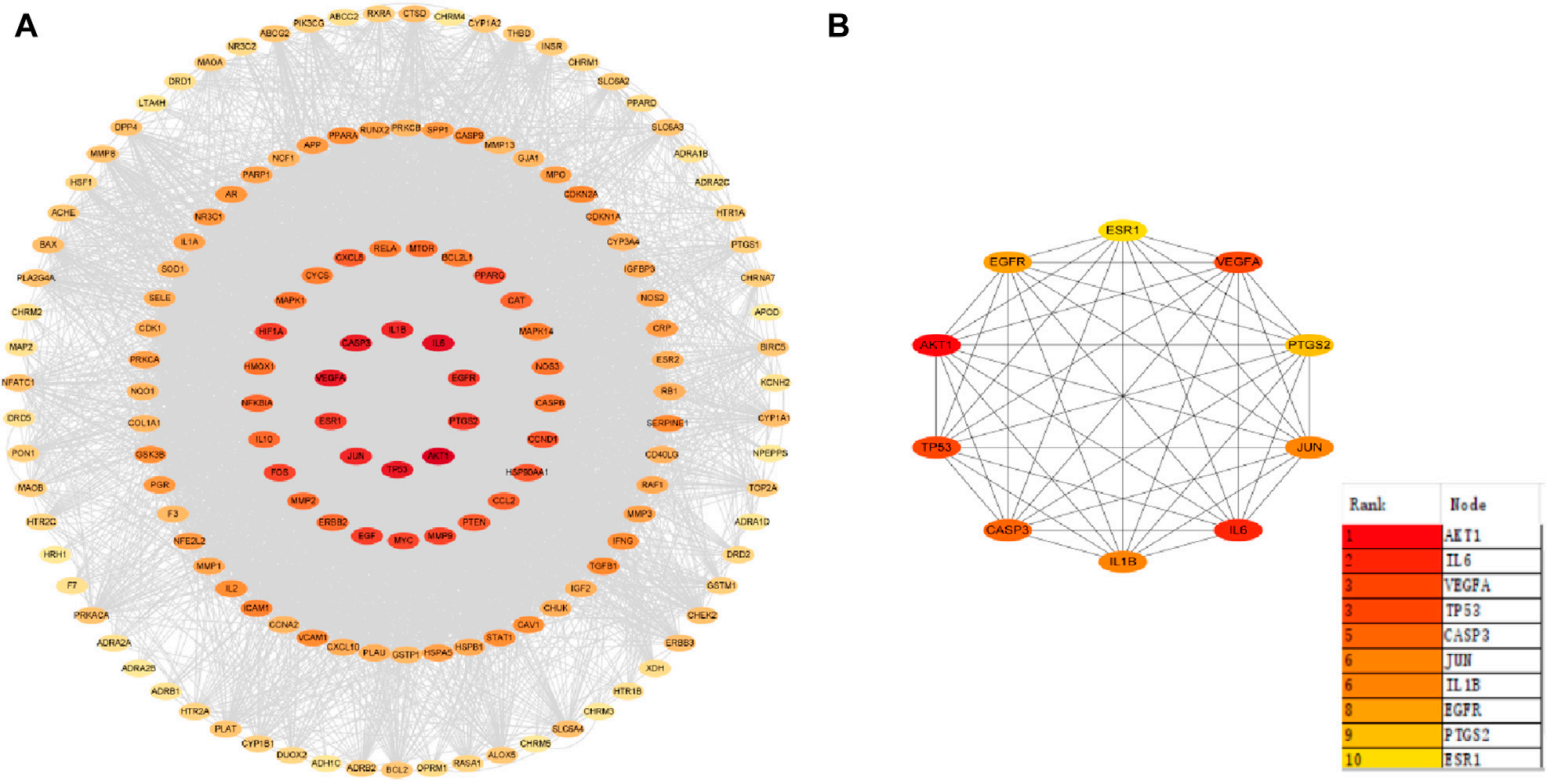
Protein-protein interaction network. **(A)** Core target co-occurrence map. **(B)** Core target interaction map.

Herbal ingredients were analyzed in the STRING for PPI with AD targets, and results were visualized in Cytoscape 3.9.1, generating a PPI network diagram based on indegree values (Figure 4). Of the 151 intersecting targets, 70 had a confidence score >0.95, leading to the identification of 10 core targets: Protein kinase B1 (AKT1), IL6, vascular endothelial growth factor A (VEGFA), tumor protein P53 (TP53), Caspase 3 (CASP3), JUN, IL1B, epidermal growth factor receptor (EGFR), prostaglandin G/H synthase 2 (PTGS2), and estrogen receptor 1 (ESR1). Notably, components diosgenin and baicalein from *Polygonatum sibiricum Redout*, along with quercetin and beta-sitosterol from *Lycium chinense Mill*, were found to interact with these core targets.

### 3.4 Molecular docking analysis

**FIGURE 5:**
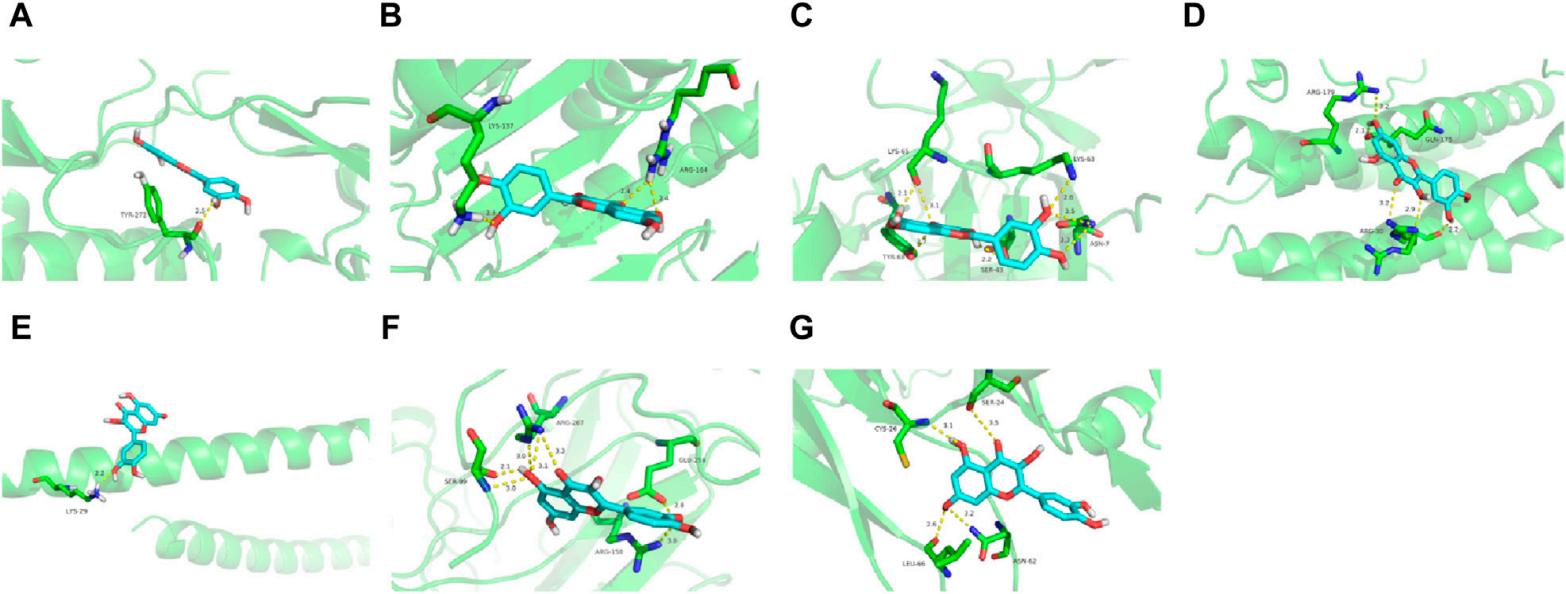
Quercetin docking with core target proteins. **(A)** AKT1 **(B)** CASP3 **(C)** IL1B **(D)** IL6 **(E)** JUN **(F)** TP53 **(G)** VEGFA.

To further assess EJW’s effects on AD, components including diosgenin, baicalein, beta-sitosterol, and quercetin were docked with core target proteins (JUN, AKT1, IL1B, CASP3, VEGFA, TP53, and IL6). Ligand-receptor binding stability and binding energy were inversely correlated. Binding energy below −5.0 kcal mol^-1^ indicates effective interaction, and below −7.0 kcal mol^-1^ suggests strong binding activity. Our findings reveal that the binding energies of these components with core targets are all < -5.0 kcal mol^-1^, demonstrating strong binding through hydrogen bonding. Quercetin, in particular, showed the highest binding energy (−9.9 kcal mol^-1^) with AKT1, and diosgenin showed the lowest (−5.3 kcal mol^-1^) with IL6. Representative molecular docking of quercetin with core targets is illustrated in Figure 5.

### 3.5 GO and KEGG analyses were performed

**FIGURE 6:**
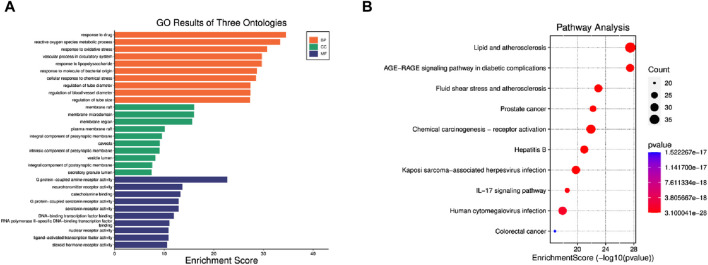
GO and KEGG enrichment analysis. **(A)** GO enrichment analysis. **(B)** KEGG enrichment analysis.

To delve deeper into EJW’s specific mechanisms for treating AD, we input EJW components and AD common targets into the DAVID database. Utilizing *p* < 0.05 as the threshold, R language was employed to generate GO and KEGG enrichment analysis graphs for the top 10 ranked entries. The GO enrichment analysis identified 2929 entries, with 2607 related to BP, predominantly involving drug response, reactive oxygen species metabolic process, and oxidative stress response. A total of 107 entries were associated with CC, mainly membrane raft, membrane microdomain, and membrane region. Additionally, 215 entries were related to MF, including G protein-coupled amine receptor activity, neurotransmitter receptor activity, and catecholamine binding (Figure 6A). The KEGG enrichment analysis revealed 180 pathways, notably lipid metabolism and atherosclerosis, AGE-RAGE signaling in diabetic complications, and fluid shear stress in atherosclerosis (Figure 6B).

### 3.6 Effects of EJW on learning and memory ability in APP/PS1 mice

**FIGURE 7:**
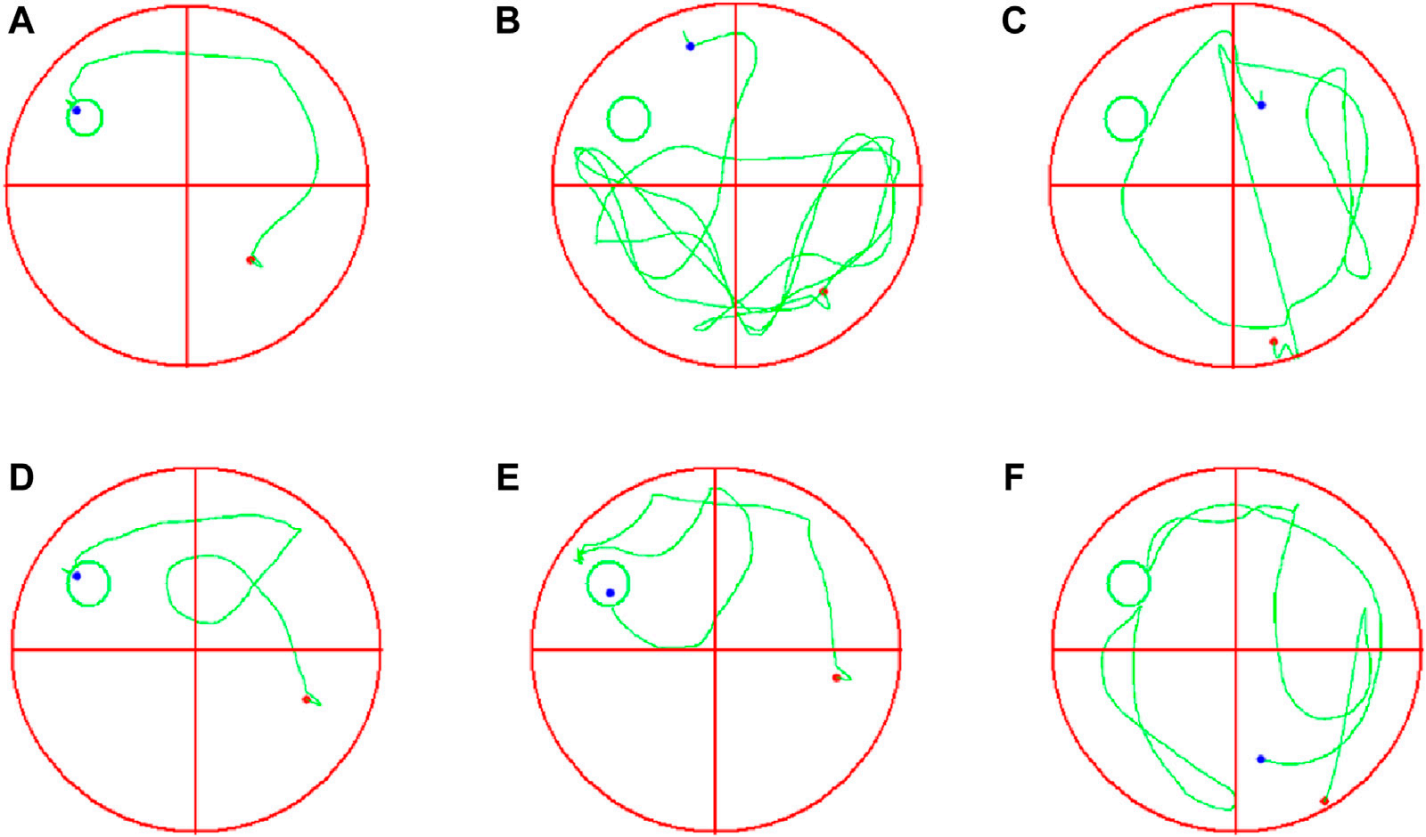
Morris water maze navigation tracks for each mouse group. **(A)** Blank group mice displayed short, efficient navigation paths, quickly locating the platform. **(B)** Model group mice showed longer, more erratic paths without locating the platform within the allotted time. **(C)** Control group mice had shorter paths than the model group but failed to locate the platform. **(D)** EJW-H group mice exhibited reduced navigation distances before finding the platform. **(E)** EJW-M group mice located the platform, albeit with longer distances than EJW-H group mice. **(F)** EJW-L group mice had long navigation distances with unclear paths, failing to find the platform.

**FIGURE 8:**
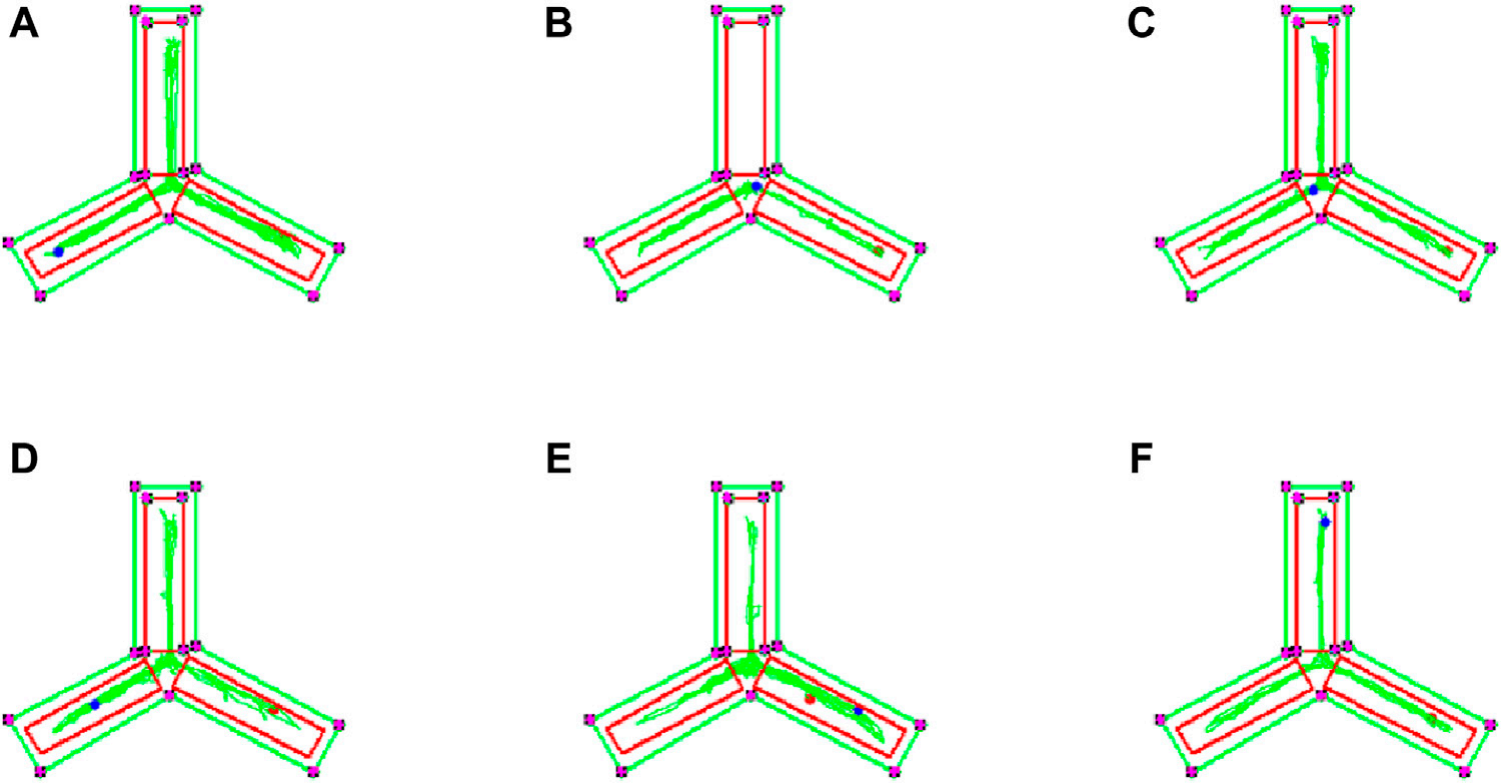
Y maze navigation tracks of each mouse group. **(A)** Mice in the blank group exhibited longer exploration distances and entered the novel arm more frequently. **(B)** In contrast to the blank group, mice in the model group showed reduced exploration distances and did not enter the novel arm. **(C)** Compared to the model group, mice in the control group entered the novel arm with more distinct navigation paths. **(D)** Similarly, mice in the EJW-H group explored the novel arm and demonstrated clearer tracks. **(E)** While mice in the EJW-M group also entered the novel arm, their exploration distance was shorter than that of the EJW-H group. **(F)** Mice in the EJW-L group entered the novel arm, but their exploration paths were less defined.

**FIGURE 9:**
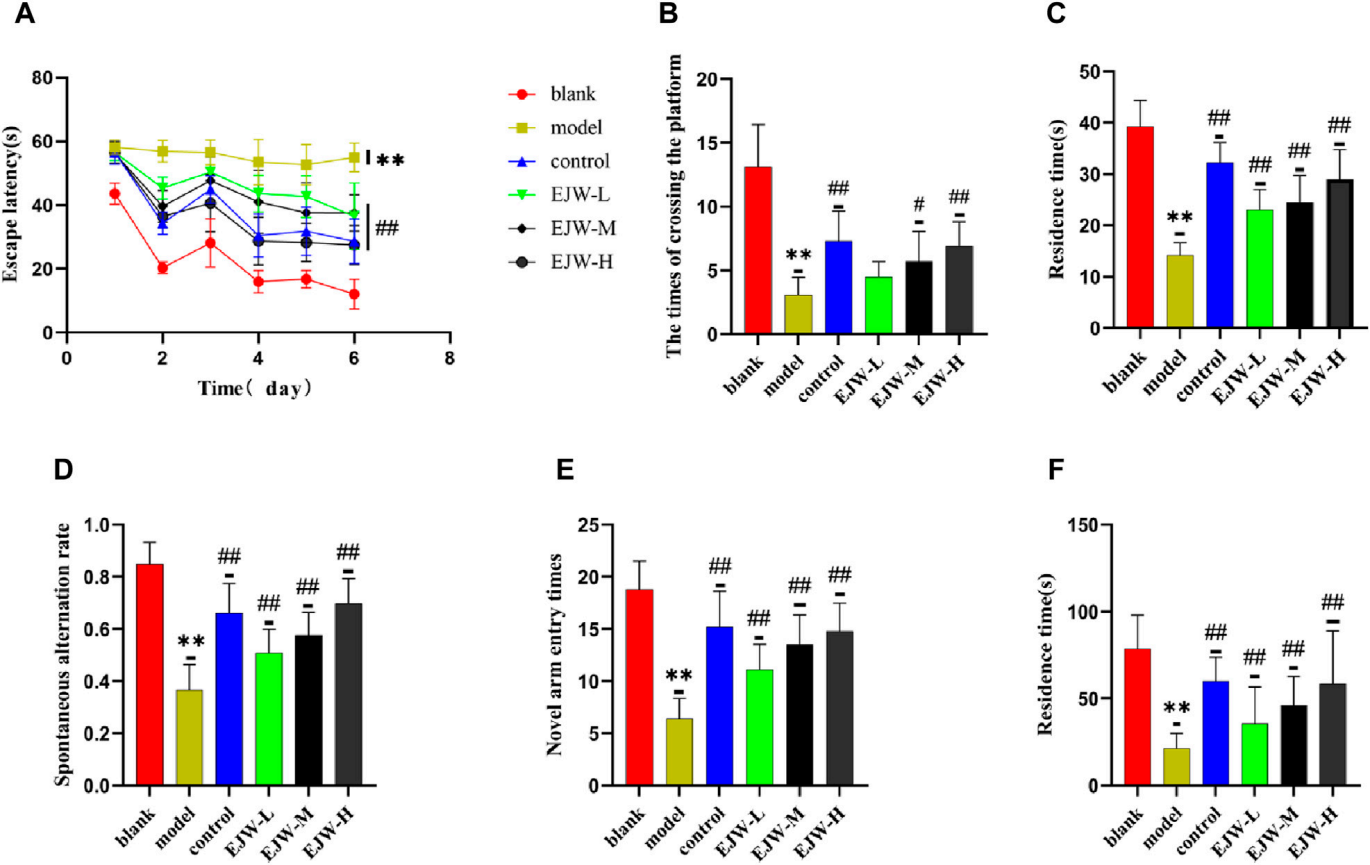
Morris water maze and Y-maze behavioral experiments results for each group of mice. **(A)** Mice in the blank group exhibited significantly lower escape latencies over the 5-day period, whereas mice in the model group experienced a marked increase in escape latency. In comparison, both the control and EJW-treated mice showed significant reductions in escape latency. **(B)** The blank group mice crossed the platform most frequently. Relative to the blank group, the model group’s platform crossings significantly decreased. However, the control and EJW groups increased their platform crossings compared to the model group, with the control group surpassing the EJW group. **(C)** Upon platform removal, the blank group mice remained in the platform area the longest. Conversely, the model group’s duration in the platform area was significantly shorter compared to the blank group, but Donepezil and EJW treatment prolonged the mice’s stay in this area. **(D)** In the Y maze, the blank group mice had the highest spontaneous alternation rate, which was significantly reduced in the model group. Following Donepezil and EJW intervention, the spontaneous alternation rate significantly increased in both the control and EJW groups. **(E)** The blank group mice entered the novel arm most frequently, whereas the model group mice had the fewest entries. Post-intervention, both the control and EJW groups exhibited more frequent entries into the novel arm than the model group. **(F)** The blank group mice spent the longest duration in the novel arm, but the model group mice had the shortest duration. Compared to the model group, the control and EJW groups significantly increased their stay time. Data are expressed as mean ± SD (*n* = 10). *p < 0.05 and **p < 0.01 *versus* the blank group, ^#^ *p* < 0.05 and ^##^ *p* < 0.01 *versus* the model group.

To evaluate the impact of EJW on cognitive functions in APP/PS1 mice, we conducted Morris water maze (Figures 7A–F) and Y maze (Figures 8A–F) tests to record navigational behaviors. The Morris water maze data revealed that on the initial day of formal trials, due to 1 day of adaptive training, the escape latency for the model group was significantly longer compared to the blank group (*p* < 0.01), however, the escape latency of EJW-L, EJW-M, EJW-H and control group mice decreased (*p* < 0.01). On the fifth day of the formal trials, the escape latency of the model group mice was still longer than that of the blank group (*p* < 0.01), and the times of crossings platform, retention time also decreased (*p* < 0.01). But the escape latency of EJW-L, EJW-M, EJW-H, and control group mice decreased (*p* < 0.01), and also demonstrating improved navigation efficiency, increased duration in the target quadrant, and more frequent platform crossings (*p* < 0.05) (Figures 9A–C). Similarly, the Y maze findings reflected these improvements, indicating the model group’s spontaneous alternation rate was significantly reduced compared to the blank group (*p* < 0.01), alongside fewer entries and diminished time spent in the new arm (*p* < 0.01). Treatment with EJW and Donepezil effectively mitigated these impairments (*p* < 0.01) (Figures 9D–F).

### 3.7 Effects of EJW on Aβ and nerve cell morphology in hippocampus of APP/PS1 mice

**FIGURE 10:**
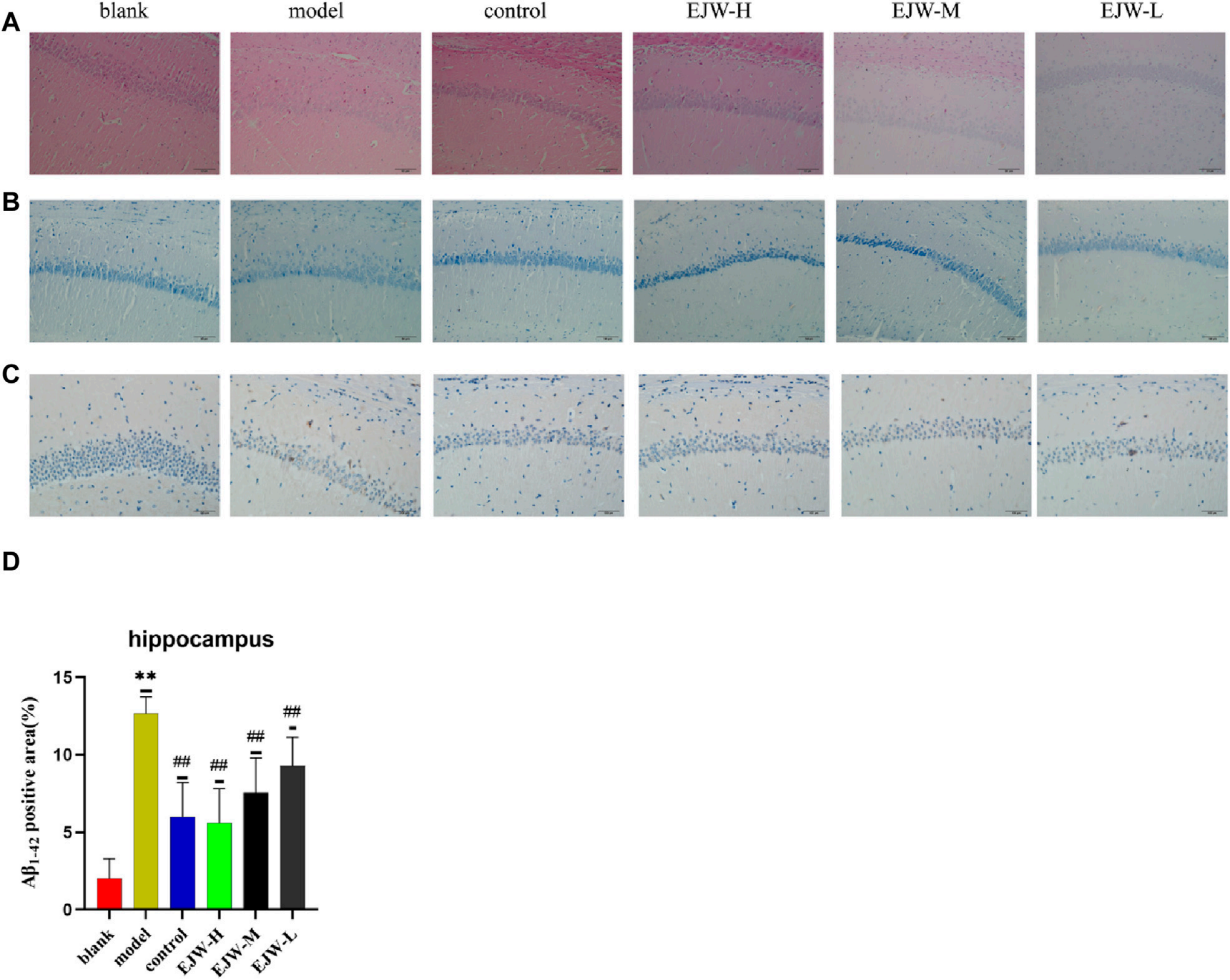
Pathological changes in the hippocampal CA1 area across groups. **(A)** HE staining revealed neat neuronal arrangement in the blank group, whereas the model group showed scattered and diminished cells, with the control and EJW groups displaying densely packed neurons. **(B)** Nissl staining highlighted prominent Nissl bodies in the blank group, absent in the model group, but significantly present in the control and EJW groups. **(C, D)** Immunohistochemical staining indicated minimal Aβ_1-42_ in the blank group, increased in the model group, but reduced in the control and EJW groups. Data are expressed as mean ± SD (*n* = 4). *p < 0.05 and **p < 0.01 *versus* the blank group, ^#^ *p* < 0.05 and ^##^ *p* < 0.01 *versus* the model group.

**FIGURE 11:**
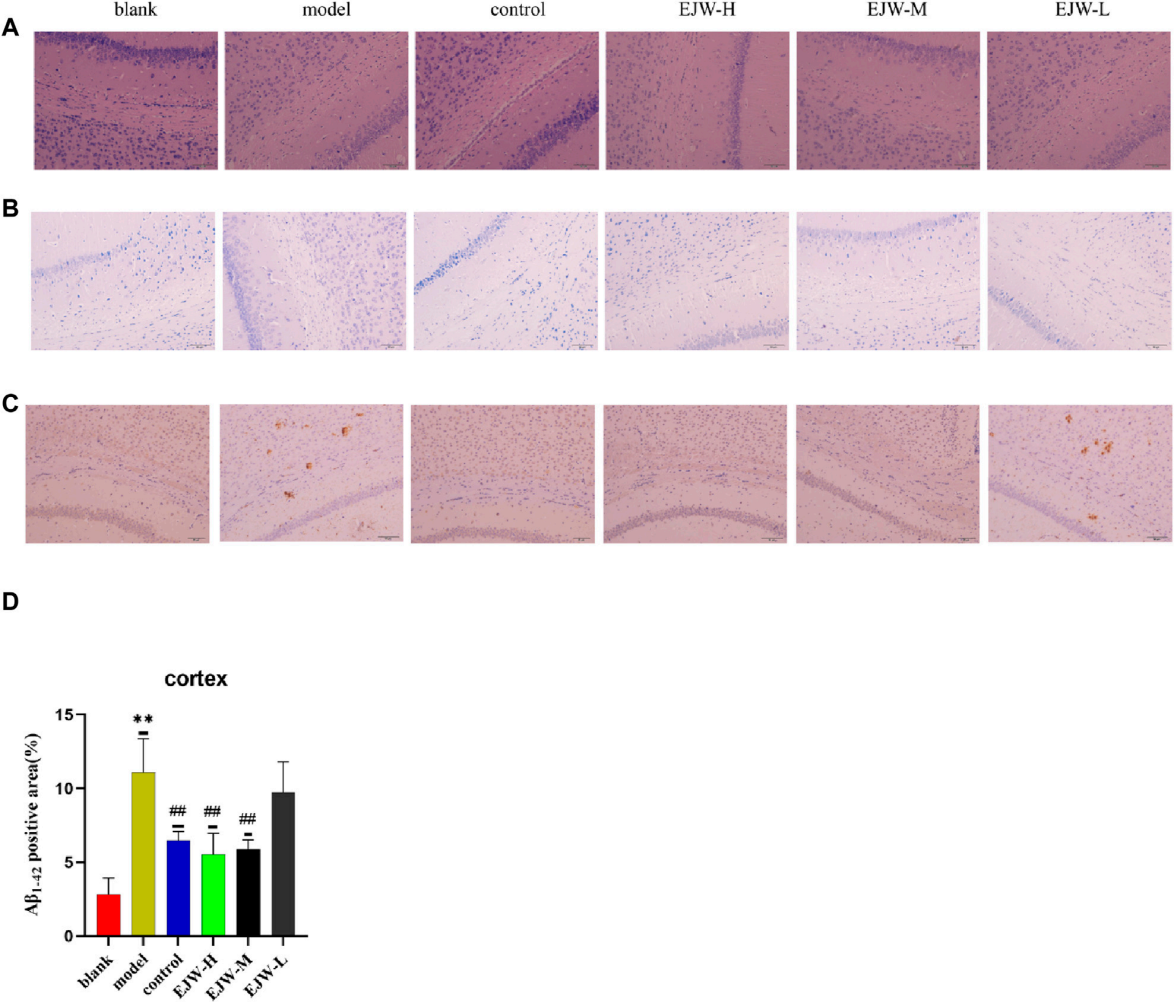
A comparison of the pathological changes in the area of the cortex in each group of mice. **(A)** HE staining showed neatly arranged neurons in the blank group, dispersed in the model group, but not observed in the control and EJW groups. **(B)** Nissl staining displayed clear Nissl bodies in the blank group, absent in the model group, but evident in the control and EJW groups. **(C, D)** Immunohistochemical results for the cortex mirrored those of the hippocampus, with the least Aβ_1-42_ in the blank group, the most in the model group, and significant reductions post-donepezil and EJW intervention. Data are expressed as mean ± SD (*n* = 4).*p < 0.05 and **p < 0.01 compared to the blank group, ^#^ *p* < 0.05 and ^##^ *p* < 0.01 compared to the model group.

To further explore EJW’s impact on the morphology of hippocampal neurons in APP/PS1 mice, HE and Nissl staining, along with IHC to assess Aβ expression, were performed. In the blank group, neurons within the hippocampal region were well-organized, with abundant cells, clearly defined nuclei, robust cytoplasm, high density of Nissl bodies, and no significant Aβ plaque accumulation. In contrast, the model group’s hippocampal neurons appeared disorganized, with reduced Nissl bodies and neuron numbers, and extensive Aβ plaque deposition. Post-EJW treatment, the hippocampal neuronal morphology largely returned to normal, with an increase in Nissl bodies and a marked decrease in Aβ plaque deposition, mirroring behavioral test outcomes (Figure 10). While EJW improved hippocampal neural cell morphology and reduced Aβ plaques in APP/PS1 mice, its effects on the cerebral cortex pathology remained uncertain. Nonetheless, EJW also reduced cortical Aβ plaques and enhanced Nissl body and neuron numbers. Compared to the model group, however, the EJW-L group showed no significant difference in Aβ plaque area (Figure 11).

### 3.8 Effects of EJW on blood lipid and blood glucose levels in APP/PS1 mice

**FIGURE 12:**
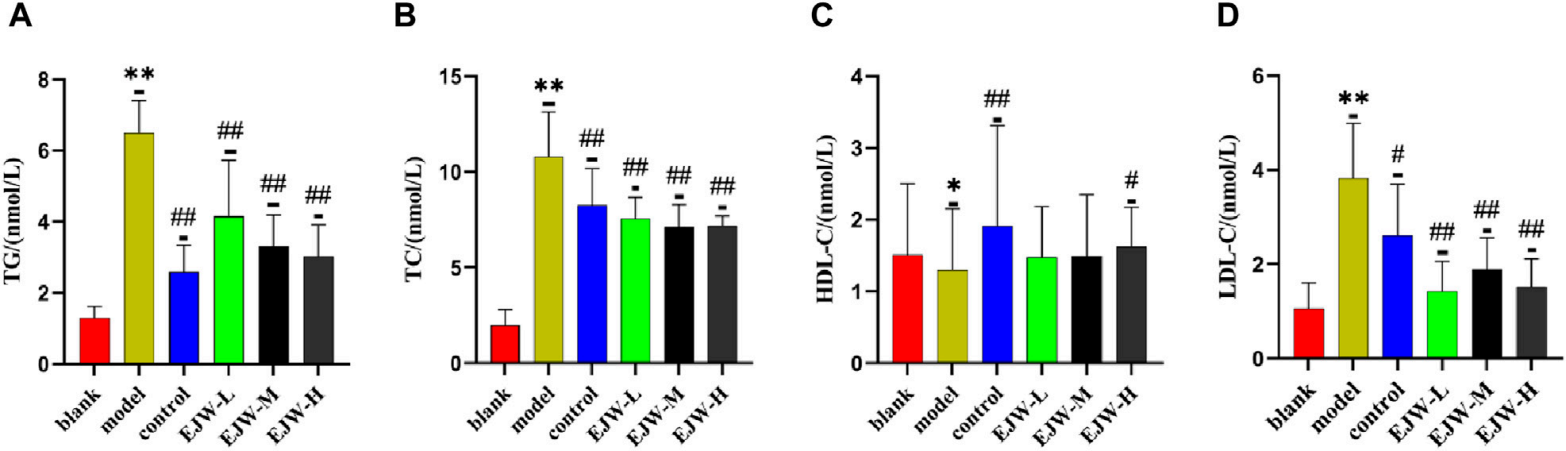
Biochemical analysis of mouse serum. **(A)** TG levels were lowest in the blank group, significantly higher in the model group, but reduced following EJW and Donepezil intervention. **(B)** TC levels followed a similar pattern, with the model group showing a significant increase compared to the blank group, which was mitigated by EJW and Donepezil treatment. **(C)** HDL-C levels were highest in the blank group, significantly decreased in the model group, but increased after treatment, with no marked difference in the EJW-M and EJW-L groups. **(D)** LDL-C levels were lowest in the blank group, significantly increased in the model group, but decreased following treatment. Data are presented as mean ± SD (*n* = 6).*p < 0.05 and **p < 0.01 compared to the blank group, ^#^ *p* < 0.05 and ^##^ *p* < 0.01 compared to the model group.

We examined the blood lipid profiles of the mice across all groups to assess the correlation between hippocampal pathology, blood lipid levels, and cognitive functions. Our findings revealed that, relative to the blank group, levels of TG, TC, and LDL-C in the model group’s plasma were significantly elevated, while HDL-C levels were significantly reduced. In contrast, EJW and Donepezil treatments resulted in a significant reduction in TG, TC, and LDL-C levels, and a significant increase in HDL-C levels compared to the model group (Figure 12). These outcomes align with prior behavioral and pathological results, suggesting a direct link between alterations in blood lipid levels and the accumulation of Aβ, neuronal structural disruptions, and cognitive performance.

### 3.9 Effects of EJW on SOD, ROS, IL-1β and IL-6 in the hippocampus of APP/PS1 mice

**FIGURE 13:**
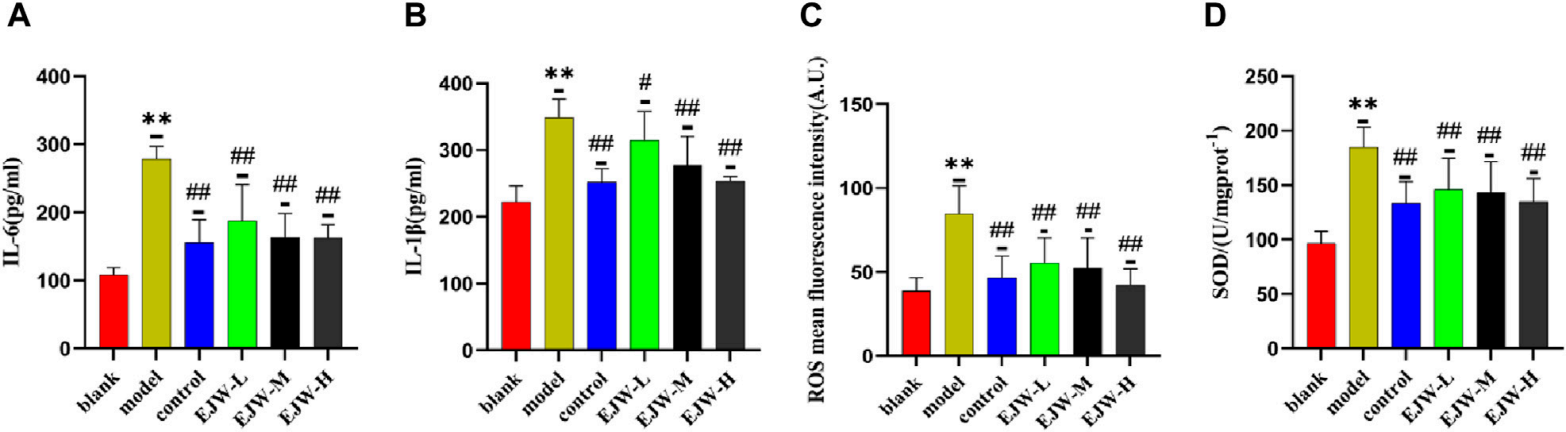
Detection of inflammation and oxidative stress markers in mouse brains. **(A, B)** In the blank group, brain levels of the inflammatory markers IL-6 and IL-1β were the lowest. In comparison, these levels were significantly elevated in the model group but notably reduced following EJW and Donepezil treatment. **(C, D)** Relative to the blank group, the model group exhibited increased ROS and decreased SOD levels in the brain, which were normalized after EJW and Donepezil intervention. Data are presented as mean ± SD (*n* = 6).**p < 0.01 compared to the blank group, ^#^ *p* < 0.05 and ^##^ *p* < 0.01 compared to the model group.

The alteration in blood lipid levels may influence antioxidant capabilities and inflammation. Consequently, we measured the expression levels of SOD, ROS, IL-1β, and IL-6 in the mice’s brains. As anticipated, compared to the blank group, ROS, IL-1β, and IL-6 levels were elevated, while SOD levels decreased in the model group’s brains. However, EJW treatment led to not only reduced levels of ROS, IL-1β, and IL-6 but also increased SOD levels compared to the model group (Figure 13). This suggests that changes in blood lipid levels may contribute to heightened inflammation and oxidative stress in the brain.

### 3.10 Effects of EJW on APOA-I, APOB, APOE4, AGEs, RAGE and NF-κB mRNA in hippocampus of APP/PS1 mice

**FIGURE 14:**
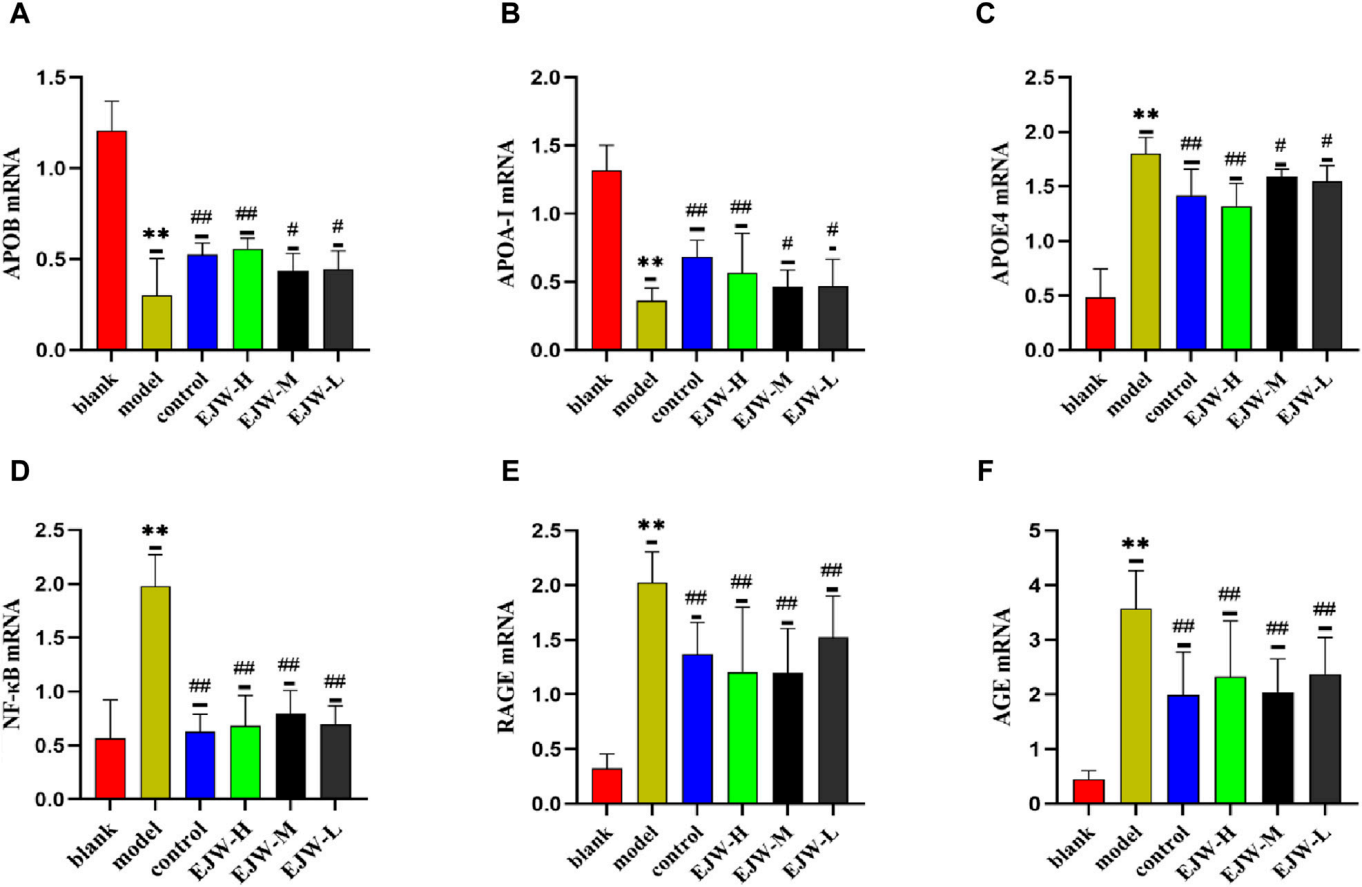
mRNA detection of apolipoprotein and AGEs/RAGE/NF-κB pathway in the brains of mice in each group. **(A–C)** Compared to the blank group. Post-EJW and Donepezil treatment, APOE4 mRNA decreased, whereas APOA-I and APOB mRNA levels increased. **(D–F)** AGE, RAGE, and NF-κB mRNA levels increased in the model group compared to the blank group but normalized following EJW and Donepezil intervention. Data are presented as mean ± SD (*n* = 6).**p < 0.01 compared to the blank group, ^#^ *p* < 0.05 and ^##^ *p* < 0.01 compared to the model group.

APOA-I, APOB, and APOE4 are integral to lipid synthesis, transport, and degradation, significantly influencing blood lipid levels. Following observations of abnormal blood lipid levels in mouse plasma, we investigated the mRNA levels of APOA-I, APOB, and APOE4 in the mice’s brains. The results indicated a significant increase in APOE4 mRNA expression in the model group, while APOA-I and APOB mRNA expression levels decreased. Conversely, EJW treatment reversed these trends (Figures 14A–C). This suggests that altered blood lipid levels in mice may result from lipoprotein imbalances, affecting lipid transport and degradation.

To further understand how EJW improves AD symptoms at the lipid level, we examined mRNA expression of AGE, RAGE and NF-κB, linked to lipid metabolism, based on KEGG core target enrichment analysis results. Relative to the blank group, mRNA levels of AGE, RAGE, and NF-κB in the model group’s brains were significantly higher. EJW treatment reduced these levels (Figures 14D–F), suggesting EJW may mitigate AGEs/RAGE/NF-κB signaling pathway activation, enhancing lipid metabolism and thereby exerting a therapeutic effect on AD.

### 3.11 Effects of EJW on AGE, RAGE, NF-κB and tau in hippocampus of APP/PS1 mice

**FIGURE 15:**
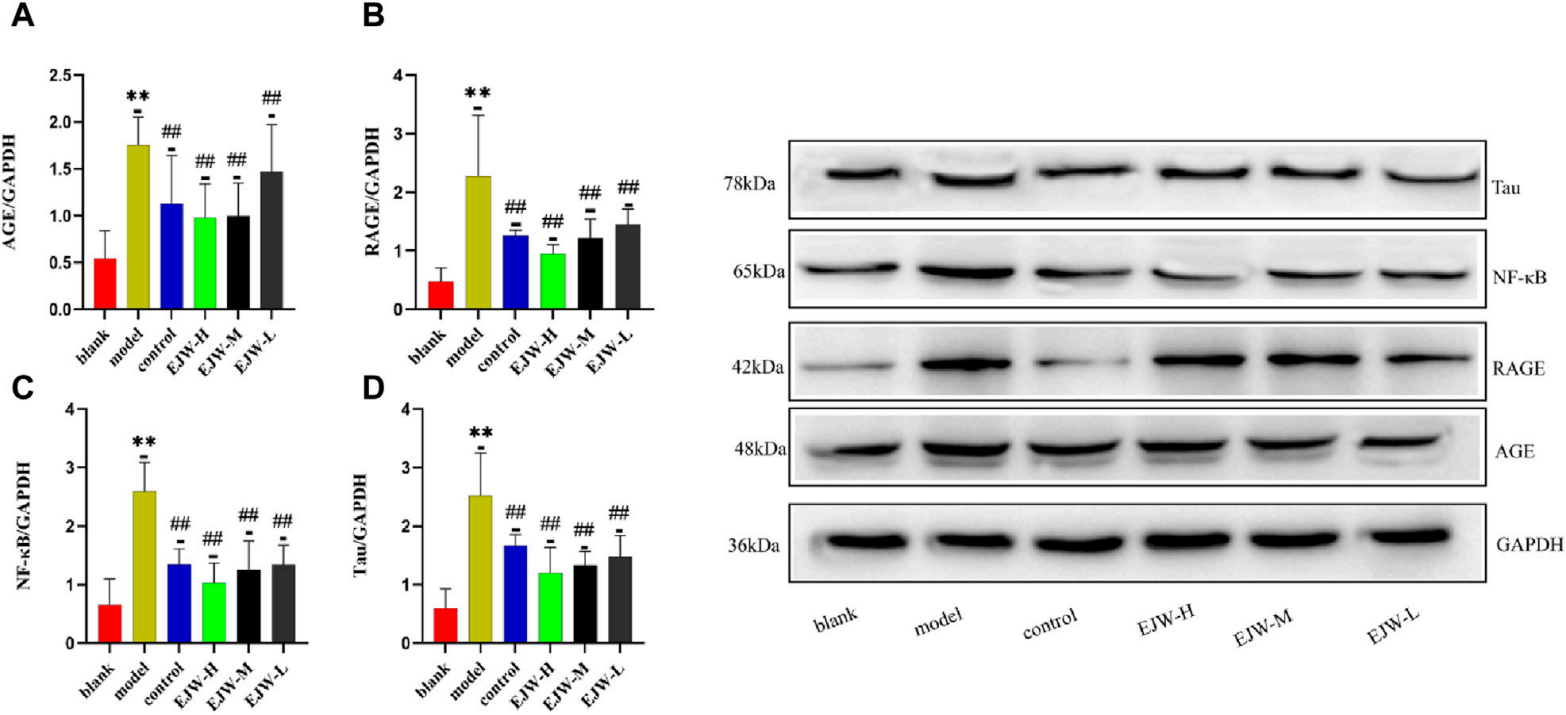
Detection of Tau and AGEs/RAGE/NF-κB pathway related proteins in mice brains. **(A–C)** Levels of AGE, RAGE, and NF-κB proteins were higher in the model group’s brain compared to the blank group but were lowered after treatment with EJW and Donepezil. **(D)** Tau protein levels increased in the model group but decreased following EJW and Donepezil intervention. Data are expressed as mean ± SD (*n* = 6).**p < 0.01 compared to the blank group, ^#^ *p* < 0.05 and ^##^ *p* < 0.01 compared to the model group.

Despite having quantitatively analyzed the mRNA levels of AGE, RAGE, and NF-κB, we had not yet assessed their transcriptional activity. To ascertain the regulatory impact of EJW on the AGEs/RAGE/NF-κB pathway, we evaluated the protein expression of AGE, RAGE, and NF-κB. Relative to the blank group, the model group exhibited a significant increase in the hippocampal levels of AGE, which was markedly reduced by EJW treatment, a trend also observed in the control group (Figure 15A). Similarly, the levels of RAGE and NF-κB proteins in the hippocampus were significantly higher in the model group compared to the blank group but were diminished following EJW intervention (Figures 15B,C). Given Tau’s role as a key AD pathology marker and its interaction with the AGEs/RAGE/NF-κB pathway, we also measured Tau protein expression. Tau levels were notably elevated in the hippocampus of model mice compared to the blank group but were reduced by EJW treatment (Figure 15D).

## 4 Discussion

The traditional Chinese medicine formula EJW, originating from the ancient text “Sheng Ji Zong Lu” during the Song Dynasty, has been explored for its therapeutic effects on Alzheimer’s Disease (AD) with its two key ingredients: *Polygonatum sibiricum Redout* and *Lycium chinense Mill*. Yet, the precise mechanisms by which EJW exerts its effects in AD treatment remain to be fully understood. To address this, our study utilized network pharmacology to identify the herbal components and their associated targets within EJW, and to pinpoint AD-related targets across multiple disease databases. This approach led to the identification of potential targets for EJW in AD treatment and clarification of its specific action mechanisms via GO and KEGG enrichment analyses. The efficacy of these mechanisms was then validated through *in vivo* animal studies.

APP/PS1 mice, a commonly used and reliable double transgenic model for AD research, express mutated forms of the β-amyloid precursor protein (APP swe) and human presenilin one gene (PS1246E), which result in elevated levels and aggregation rates of Aβ protein in the brain, mirroring the plaque formation seen in human AD (Porquet et al., 2015; Chen and Zhang, 2022). Cognitive impairments, particularly in learning and memory, become evident as early as 3 months of age in these mice, with progressive worsening by 6 months (Lok et al., 2013). Our experimental findings from the Morris water maze and Y-maze tests indicated that APP/PS1 mice demonstrated significantly increased escape latencies, reduced time in target quadrants, and fewer platform crossings. Furthermore, these mice displayed a lower spontaneous alternation rate, reduced time in the novel arm, and fewer entries. However, APP/PS1 mice treated with EJW showed marked improvements in these areas, suggesting EJW’s potential to enhance spatial working memory and reference memory in APP/PS1 mice.

When excessive Aβ accumulates in the brain, its neurotoxic effects can cause nerve cell damage and loss, alongside a decrease in Nissl bodies, resulting in impaired memory and learning. Studies have shown that intracerebral injection of Aβ_25-35_ reduces nerve cell and Nissl body counts in rats, elevates acetylcholinesterase (AchE) levels, and markedly diminishes learning and memory capabilities (Du et al., 2020). Similarly, elevated Aβ levels in APP/PS1 mice brains lead to neuron and synapse reduction and increased oxidative stress (Wang et al., 2011). In our study, a notable decline in neuron and synapse numbers in the hippocampus of APP/PS1 mice was observed, accompanied by nuclear loss, reduced cytoplasm, and heightened Aβ plaque accumulation. Conversely, EJW preserved neuronal morphology in the hippocampus and lowered Aβ plaque deposition, aligning with results from the Y-maze and Morris water maze tests. These findings suggest EJW’s potential to mitigate Aβ accumulation, safeguard neuronal integrity, and enhance cognitive functions in mice.

Dysfunction in lipid metabolism can lead to various diseases, including atherosclerosis, diabetes, and hyperlipidemia. Recent studies have demonstrated that impaired lipid transport and degradation can impact cognition and memory, potentially contributing to the development of AD. Notably, lipid-lowering medications such as lovastatin and atorvastatin have shown efficacy in ameliorating AD clinical symptoms (Ben-Aicha et al., 2020; Song et al., 2021; Ezkurdia et al., 2023). Lipid cholesterol is crucial for cell membrane stability and is found in blood lipoproteins such as HDL, LDL, VLDL, and chylomicrons. These lipoproteins interact with apolipoproteins to regulate cholesterol homeostasis in the central nervous system (CNS) (Hottman et al., 2014). APOA-I facilitates the efflux of cholesterol and phospholipids from cells, whereas APOB48 and APOB100 are involved in the cellular uptake of triglycerides and cholesterol, leading to potential cholesterol accumulation and increased blood-brain barrier (BBB) permeability. This facilitates the entry of lipoproteins and Aβ into the CNS. Studies have indicated that low HDL levels and high cholesterol may contribute to cognitive decline, though findings in this area are mixed (Tynkkynen et al., 2016; de Dios et al., 2023). Notably, APOE is instrumental in transporting cholesterol and phospholipids within the brain, with its APOE4 variant binding preferentially to LDL and VLDL, thus elevating CNS cholesterol levels. Such an environment promotes Aβ generation and aggregation, as high cholesterol influences the APP processing pathway, enhancing β-secretase and γ-secretase activity (Di Paolo and Kim, 2011). Furthermore, the APOE4 allele, a significant risk factor for AD, exhibits a higher affinity for Aβ. Complexes formed between APOE4 and Aβ exhibit a reduced internalization rate due to clearance by the Very Low Density Lipoprotein Receptor (VLDLR) alone, as opposed to additional clearance by LRP1 and VLDLR at the BBB, leading to Aβ plaque accumulation (Deane et al., 2008; Holtzman et al., 2012). Our research indicates that EJW significantly decreases TG, TC, and LDL levels in APP/PS1 mice plasma, increases HDL levels, and reduces APOB and APOE4 mRNA expression while enhancing APOA-I mRNA expression. These findings align with behavioral and pathological observations, suggesting EJW’s potential in improving lipid metabolism, reducing Aβ deposition, and enhancing learning and memory in APP/PS1 mice.

AGE are generated through non-enzymatic reactions between amino groups in amino acids or proteins and reduced sugar carbonyl groups, as well as through sugar oxidation under oxidative stress (Gill et al., 2019; Reddy et al., 2022). AGEs can bind to various cell surface receptors, notably the receptor for advanced glycation end products (RAGE), a pattern recognition receptor (Neeper et al., 1992; Cai et al., 2018). The AGE-RAGE complex activates multiple signaling pathways, including mitogen-activated protein kinases (MAPKs), Janus kinases (JAK), phosphoinositide 3-kinase (PI3K), and the NF-κB pathway. NF-κB, a transcription factor, induces the expression of pro-inflammatory genes and activates inflammatory cytokines (IL-1, IL-6, and TNF-α). Interestingly, RAGE can also facilitate the transport of Aβ across the blood-brain barrier (BBB) into the brain. Additionally, AGE-RAGE binding triggers the activation of nicotinamide adenine dinucleotide phosphate (NADPH) oxidase, leading to increased production of reactive oxygen species (ROS), malondialdehyde (MDA), and superoxide anion, thus exacerbating oxidative stress and AGE levels (Reddy et al., 2023). Studies have shown that overexpression of proteins in the AGEs/RAGE/NF-κB pathway in the brains of SD rats injected with lipopolysaccharide (LPS) results in elevated levels of IL-1β, IL-6, and TNF-α, alongside increased oxidative stress (Li et al., 2022). Another study highlighted the activation of the AGEs/RAGE/NF-κB pathway and mitochondrial dysfunction in SH-SY5Y cells exposed to oxidative magnesium (Wang et al., 2014). The scavenger receptor family, which includes class A scavenger receptor (SR-A), recognizes oxidized or acetylated low-density lipoprotein (OxLDL or AcLDL) and mediates the internalization and degradation of AGEs, competing with LDL (Araki et al., 1995). Our research on the efficacy of EJW in treating AD by regulating cholesterol metabolism involved analyzing mRNA expression in the AGEs/RAGE/NF-κB pathway, informed by network pharmacology results. We discovered that EJW reduces the expression of AGEs, RAGE, NF-κB, and the activity of IL-1β, IL-6, and ROS, while increasing superoxide dismutase (SOD) levels. This suggests EJW inhibits the AGEs/RAGE/NF-κB pathway, diminishing inflammation and oxidative stress in the brains of APP/PS1 mice, and enhancing their learning and memory capabilities. Furthermore, EJW was found to decrease Tau protein expression in the brains of APP/PS1 mice, potentially due to the high APOE4 expression leading to Aβ aggregation and subsequent Tau protein overexpression [(Gomes et al., 2019; Zhang et al., 2021b)].

## 5 Conclusion

In summary, we identified four key components of EJW for treating AD through network pharmacology: iosgenin, baicalein, beta-sitosterol, and quercetin, and we screened ten core targets, including AKT1, IL6, VEGFA, TP53, CASP3, among others. Our experimental results demonstrate that EJW can reduce TG, TC, LDL, and APOE4 mRNA levels while increasing HDL, APOA-I mRNA, and APOB mRNA levels. This indicates an improvement in cholesterol metabolism in APP/PS1 mice. Additionally, EJW inhibits the overexpression of Aβ_1-42_, Tau, IL-6, IL-1β, and ROS, increases SOD levels, and enhances learning and memory abilities in mice. The therapeutic effect of EJW on AD may be attributed to its inhibition of the AGEs/RAGE/NF-κB pathway.

## Data Availability

The original contributions presented in the study are included in the article/Supplementary material, further inquiries can be directed to the corresponding author.
